# Estimation of patient organ doses from CT examinations in Tanzania

**DOI:** 10.1120/jacmp.v7i3.2200

**Published:** 2006-08-24

**Authors:** Justin E. Ngaile, Peter K. Msaki

**Affiliations:** ^1^ Radiation Control Directorate Tanzania Atomic Energy Commission P.O. Box 743 Arusha Tanzania; ^2^ Department of Physics University of Dar es Salaam P.O. Box 35063 Dar es Salaam Tanzania

**Keywords:** patient organs doses, CT

## Abstract

Although the use of CT in medical diagnosis delivers radiation doses to patients that are higher than those from other radiological procedures, lack of optimized protocols could be an additional source of increased dose in developing countries. The aims of this study are, first, to determine the magnitude of radiation doses received by selected radiosensitive organs of patients undergoing CT examinations and compare them with other studies, and second, to assess how CT scanning protocols in practice affect patient organ doses. In order to achieve these objectives, patient organ doses from five common CT examinations were obtained from eight hospitals in Tanzania. The patient organ doses were estimated using measurements of CT dose indexes (CTDI), exposure‐related parameters, and the ImPACT spreadsheet based on NRPB conversion factors. A large variation of mean organ doses among hospitals was observed for similar CT examinations. These variations largely originated from different CT scanning protocols used in different hospitals and scanner type. The mean organ doses in this study for the eye lens (for head), thyroid (for chest), breast (for chest), stomach (for abdomen), and ovary (for pelvis) were 63.9 mGy, 12.3 mGy, 26.1 mGy, 35.6 mGy, and 24.0 mGy, respectively. These values were mostly comparable to and slightly higher than the values of organ doses reported from the literature for the United Kingdom, Japan, Germany, Norway, and the Netherlands. It was concluded that patient organ doses could be substantially minimized through careful selection of scanning parameters based on clinical indications of study, patient size, and body region being examined. Additional dose reduction to superficial organs would require the use of shielding materials.

PACS numbers: 87.59 Fm; 87.66Jj; 87.52‐g

## I. INTRODUCTION

It is well known that patient doses from CT procedures are relatively higher than doses from other imaging modalities based on ionizing radiation. For example, one CT examination of the chest delivers about 400 times the dose delivered by a conventional chest X‐ray examination.^(^ ^1^ ^,^ ^2^ ^)^ Therefore, although CT represents only 5% of the total number of medical X‐ray procedures worldwide, this high dose procedure contributes about 34% of the annual collective dose from all medical X‐ray examinations to the population.^(1)^ This contribution is inevitable because it results from a combination of high dose per examination and frequent use of CT examination in diagnosis.^(^ ^1^ ^–^ ^3^ ^)^ This trend has started to appear in Tanzania, where use of CT for medical examination started as early as 1996. Currently, there are about 12 CT scanners in the country, and nearly 10 000 X‐ray CT examinations are now performed annually. This constitutes about 1% of all medical X‐ray procedures performed in Tanzania.^(^ ^1^ ^,^ ^4^ ^)^

Increased use of this high dose procedure has been of great concern globally because of the high possibility of inducing undesired health effects, such as induction of cancer, in patients.^(5)^ Of prime concern is the significant radiation dose delivered to superficial radiosensitive organs such as the eye lens, breast, and thyroid, which are, unfortunately, irradiated during radiological procedures of the head, chest, and cervical spine.^(^ ^3^ ^,^ ^6^ ^,^ ^7^ ^)^ The implication of some of these exposures, for example, to the breast and eyes, is the potential increase in the risk of breast cancer and cataract formation in the population.^(^ ^7^ ^,^ ^8^ ^)^ Since radiation exposure of different organs leads to different health effects, it has been of interest in this work to determine the actual doses delivered to individual organs.

The most useful way to assess organ doses is either by direct measurement (on patients using thermoluminescent dosimeters (TLDs) or on phantom using either an ionization chamber or TLDs) or by indirect measurement through measurement of CT dose indexes (CTDI) and published conventional factors obtained from Monte Carlo simulation and mathematical phantoms.^(^ ^6^ ^,^ ^9^ ^)^ Since dose measurement using TLDs is laborious and time‐consuming for a wide survey, the patient organ doses in this study were determined using measurements of CTDI and published conversion factors. With the knowledge of organ doses it would be easy to identify the organs at greatest radiation risk, which requires immediate protective measures. In addition, countries with insufficient experience in the use of this modality may have additional sources of increased dose to patients, such as lack of optimized techniques.^(2)^ The aims of this study, therefore, are to assess the magnitude of organ dose imparted to patients undergoing CT examinations in Tanzania and to compare them with other studies from developed countries, and to assess the effect of CT scanning protocols on patient doses.

## II. MATERIALS AND METHODS

### A. Data collection

The data used in this study were collected from eight hospitals in Tanzania with CT scanners: Muhimbili National Hospital (MNH), Kilimanjaro Christian Medical Centre (KCMC), Bugando Medical Centre (BMC), the Agha Khan Hospital (AGKH), TMJ Hospital (TMJH), the Mission Mikocheni Hospital (MMH), Regency Medical Centre (RMC), and Shree Hindu Mandal Hospital (SHMH). Detailed specifications of the scanner used at each of these hospitals are contained in Table 1. Patient data collected from these centers were further classified in two categories. In the first, data were collected to study the effects of patient‐related parameters (e.g., age, sex, diagnostic purpose of examination, body region, and use of contrast media) on organ dose. In the second, data were collected to investigate the effect of exposure‐related parameters (gantry tilt, kilovoltage (kV), tube current (mA), exposure time, slice thickness, table increment, number of slices, and start and end positions of scans) on organ dose. The selected investigations used in this study represented over 90% of the total CT examinations conducted in Tanzania today. The collection of patient exposure parameters was done using patient dose survey forms prepared for collection of patient exposure‐related parameters, hospital, and scanner used in each hospital. This form also includes a diagram of the human skeleton, on which the reported upper and lower extent of each scan was marked. A minimum number of 10 patients were used for each selected CT examination per hospital. The sum of 191, 74, 84, 88, and 54 (for 6 hospitals only) CT examinations of head, chest, abdomen, lumbar spine, and pelvis, respectively, was collected, making a total collection of 491 (about 500) examinations.

**Table 1:** Specifications of CT scanners used at each hospital in Tanzania

Hospital	Manufacturer	Scanner model/Scan mode	Focal axial distance (FAD)/Detector type
MNH	Philips Medical Systems manufactured: 1995 installed: 1996	Tomoscan SR 4000 single slice, axial and helical modes	FAD: 606 mm Detector: xenon gas
KCMC	Philips Medical Systems manufactured: 1995 installed: 1996	Tomoscan SR 4000
single slice, axial and helical modes	FAD: 606 mm detector: xenon gas
RMC	Philips Medical Systems manufactured: 1996 installed: 1999	Tomoscan M‐EG single slice, axial and helical modes	FAD: 480 mm detector: solid state
BMC	Philips Medical Systems manufactured: 2000 installed: 2001	Tomoscan M‐EG single slice, axial and helical modes	FAD: 480 mm detector: solid state
TMJH	Siemens Medical Systems manufactured: 1999 installed: 2002	Somatom Plus 4 single slice, axial and helical modes	FAD: 570 mm detector: xenon gas
MMH	Siemens Medical Systems manufactured: 1998 installed: 1999	Somatom AR. Star single slice axial and helical modes	FAD: 510 mm detector: xenon gas
AGKH	GE Medical Systems manufactured: 2001 installed: 2003	CT/e single slice, axial and helical modes	FAD: 525 mm detector: solid state
SHMH	GE Medical Systems manufactured: installed: 2002	CT Max 640 single slice, axial mode only	FAD: 525 mm detector: solid state

### B. CT dose measurements

The patient dose estimation from CT examination using the Monte Carlo technique requires measurements of CTDI and conversion coefficient data packages.^(^ ^9^ ^–^ ^11^ ^)^ In theory, the CTDI, which is a measure of the dose from single‐slice irradiation, is defined as the integral along a line parallel to the axis of rotation (*z*) of the dose profile, *D*(z), divided by the nominal slice thickness, *t*.^(^ ^9^ ^,^ ^11^ ^–^ ^13^ ^)^ In this study, CTDI was obtained from a measurement of dose, *D*(*z*), along the *z*‐axis made in air using a special pencil‐shaped ionization chamber (Diados, type M30009, PTW‐Freiburg) connected to an electrometer (Diados, type 11003, PTW‐Freiburg). The calibration of the ion chamber is traceable to the standards of the German National Laboratory and was calibrated according to the International Electrical Commission standards.^(14)^ The overall accuracy of ionization chamber measurements was estimated to be ±5%. Measurements of CTDI in air $\left({\text{CTDI}}_{100,\text{air}}\right)$ were made as recommended by the EUR 16262EN based on each combination of typical scanning parameters obtained from each hospital.^(11)^ Unfortunately, the ${\text{CTDI}}_{100,\text{air}}$ for CT scanner model Tomoscan M‐EG from RMC were not determined due to a malfunction of the scanner. Hence, the required organ doses for this particular hospital were estimated using normalized CTDI values published by the ImPACT group.^(15)^ For the sake of simplicity, the ${\text{CTDI}}_{100,\text{air}}$ will henceforth be abbreviated as ${\text{CTDI}}_{\text{air}}$.

### C. Organ dose determinations

Doses to selected organs (e.g., eye lens, thyroid, red bone marrow, breast, lung, and ovaries) were determined using the ${\text{CTDI}}_{\text{air}}$ described in the previous section and organ dose conversion coefficients.^(16)^ Since the organ dose data are expressed in terms of absorbed dose to tissue, the measured values of dose in air ${(\text{CTDI}}_{\text{air}})$ were converted to dose to tissue ${(\text{CTDI}}_{\text{tissue}})$ using the ratio of the mass–energy absorption coefficients $\left(\mu_{\text{en}}/\rho \right)$ of tissue to air(^9^ ^,^ ^10^ ^,^ ^12^):

(1) $${\text{CTDI}}_{\text{tissue}}=\left[\frac{{\left(\mu_{\text{en}}/\rho \right)}_{\text{tissue}}}{{\left(\mu_{\text{en}}/\rho \right)}_{\text{air}}}\right]*{\text{CTDI}}_{\text{air}}.$$

Despite the fact that the mass–energy absorption coefficient depends significantly on photon energy, the ratio of the mass–energy absorption coefficient of soft tissue to air is assumed to be constant for all typical X‐ray spectra produced by the CT scanners examined with a value of 1.06 (with an error of no more than ±1%).^(^ ^10^ ^,^ ^12^ ^,^ ^15^ ^)^ By using the scanner‐specific organ dose conversion coefficient, the typical average organ dose, $D_{\text{org},\;T}$, for individual examination can be estimated by summation of the following form(^6^ ^,^ ^9^ ^,^ ^13^):

(2) $$D_{\text{org},T}={\text{CTDI}}_{\text{tissue}}\sum_{z_{1}}^{z_{2}}f(\text{organ},z),$$

where $z_{1}$ and $z_{2}$ are the start and end positions of the scanned region, respectively. The organ dose conversion factor *f* (organ, *z*) was obtained from the NRPB datasets (NRPB‐SR250) based on the Monte Carlo simulations.^(^ ^16^ ^,^ ^17^ ^)^ The CTDOSE software supplied by the ImPACT group^(15)^ was used to implement Eq. (2). The ${\text{CTDI}}_{\text{air}}$ normalized to 100 mAs ${(}_{n}{\text{CTDI}}_{\text{air}})$, CT scanner manufacturer and model, and typical scanning parameters such as kV, mA, exposure time, pitch, slice thickness, gender, and start and end positions of each scan were used as input data to the CTDOSE spreadsheet in organ dose estimations.^(^ ^10^ ^,^ ^15^ ^,^ ^16^ ^)^

**Figure 1:**
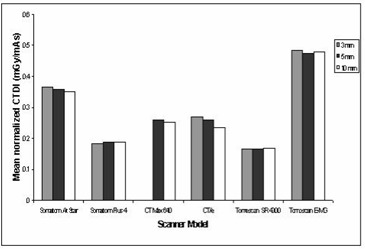
Normalized CT dose index free in air: Comparison between types of CT scanner models

**Figure 2:**
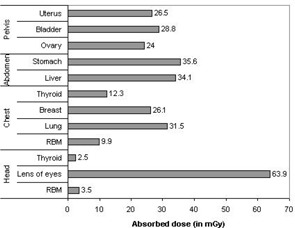
Absorbed dose for selected organs from CT examination type

Due to the fact that the software does not take into account the patient size, that is, the software was not discriminate between tall and short patients, it was necessary to adjust the scan region indicated on the human skeleton from each patient survey form in NRPB's mathematical phantom (Figs. 1 and 2 in NRPB‐R250)^(^ ^10^ ^,^ ^15^ ^)^ for each individual examination. This was done by first marking the start and end positions of the scan region and then determining the scan length from the number of slices, the slice thickness, and the table increment. This information was used in the selection of the part of the phantom irradiated in order to improve the correspondence between the organs irradiated in the patient and the phantom.^(10)^ Since the scanners used in this study (Table 1) were not in use at the time of the NRPB survey, the estimation of organ dose has to rely on the attributes of the new model compared to that of older designs. This was done using scanner‐matching data published by the ImPACT group, and may lead to uncertainty of not more than 15% of organ dose measurements.^(^ ^13^ ^,^ ^15^ ^)^ The overall uncertainties associated with estimation of organ doses, calculated by quadratic propagation of errors, were estimated to range from ±17% to ±24%. These uncertainties arose mainly from patient variations, uncertainty in the start and end positions of the scan region, calibration of the ionization chamber used to measure ${\text{CTDI}}_{\text{air}}$, statistical uncertainties in normalized organ doses, and scanner matching data. In order to evaluate how well the hospitals in Tanzania are performing in terms of minimization of organ doses associated with CT imaging, it was useful to compare mean organ dose per examination for all the hospitals that participated in the study. This was done by finding the mean organ dose from the typical patient organ dose weighted by the number of scans performed per given examination for each hospital. On the other hand, the mean value of the typical patient organ doses weighted by the number of scans per given examination, based on all hospitals in the survey, was taken as the country mean organ dose. The country mean organ doses were determined in order to compare with other studies from developed countries.

### D. Data analysis

A summary of organ dose, $D_{\text{org},\;T}$, described by Eq. (2) was estimated from about 500 CT examinations using CTDOSE software. The summary also consisted of scanning parameters (e.g., kV, mAs, slice thickness, table increment, and number of slices with and without contrast) used for each typical CT examination and CnTDIair. For each examination type per hospital, the scan parameters were registered for up to six scan sequences together with the corresponding selected organ dose. From this summary, the total organ dose for the selected organs for each examination was calculated by the summation guided by its respective scan sequences. The total organ dose for selected organ per examination, together with corresponding ${\text{CTDI}}_{\text{air}}$ and scanning parameters, was then used as input to the statistical software for analysis.

## III. RESULTS AND DISCUSSION

### A. CT scanning protocols

The mean values of scanning parameters and CnTDIair conducted in each hospital for CT examinations of (a) head, (b) chest, (c) abdomen, (d) lumbar spine, and (e) pelvis were analyzed to obtain the respective mean and related statistics. The results of the analysis are presented in Table 2. From the table, it is observed that CT scanning protocols used for a CT examination per hospital for most hospitals are highly standardized, similar to what has been experienced in other studies.^(^ ^3^ ^,^ ^17^ ^)^ For example, children, a thick patient, and a thinner patient were examined with the same exposure settings, such as kV, mA, tube rotation time (s), table increment, pitch factor, and slice thickness. Use of exposure settings depending on the size of the patient was an occasional event in a few hospitals. In clinical practice, the patient dose increases with decreasing body size.^(^ ^3^ ^,^ ^17^ ^)^ Although scanning protocols are standardized for most hospitals, it is evident from Table 2 that the standardization is not uniform across hospitals. However, the kV that is not indicated in Table 2 was an exception, since it was the same (120 kV) for all hospitals, except MMH (130 kV). The trend was almost the opposite for mAs product, which showed a large variation ranging from 60 mAs to 360 mAs for head, 60 mAs to 375 mAs for chest, 60 mAs to 355 mAs for abdomen, 60 mAs to 480 mAs for lumbar spine, and 100 mAs to 247 mAs for pelvis. In general, the lowest value was consistently used by one hospital (MMH), while the highest values varied from one hospital to another. The variation of mAs per given examination would be expected because of the difference in focus to isocenter distance among scanners. This is due to the fact that radiation intensity is inversely proportional to the square of the distance between the focus and the patient. That is, the shorter the distance, the lower the mAs values, while the longer the distance, the higher the mAs values. Of great concern in this study is the variation of mAs of up to a factor of 3.3 per examination for scanners of the same model (such as of BMC and RMC).

**Table 2:** Summary of scanning parameters and CnTDIair used by each hospital

Hospital	Exposure setting (mAs)	Slice width (mm)	Table increment (mm)	Slice No. without contrast	Slice No. with Contrast	Scan length (cm)	CnTDIair (mGy/100 mAs)
head	AGKH	247.0±26.0	5.0±0.0	6.1±0.3	23.9±2.2	23.9±2.2	22.2±11.2	27.5±1.1
	BMC	191.7±17.6	9.1±1.4	9.1±1.4	14.0±2.4	13.0±0.8	17.9±6.0	47.1±0.3
	KCMC	309.1±50.0	7.3±0.7	7.3±0.7	18.9±3.0	18.9±3.0	23.1±7.4	16.9±0.3
	MMH	275.3±32.9	3.9±0.3	3.9±0.3	32.9±6.4	32.8±6.9	24.4±9.8	36.8±0.1
	MNH	241.7±25.3	7.6±0.5	7.6±0.5	17.9±1.3	18.0±1.0	26.0±3.0	17.5±0.6
	RMC	60.0±0.0	6.3±0.4	6.3±0.4	18.9±3.1	23.9±1.4	24.2±3.7	58.4±0.0
	SHMH	275.0±0.0	7.6±0.6	7.6±0.6	17.1±1.0	17.1±1.0	24.9±4.2	27.2±2.3
	TMJH	360.0±0.0	6.1±0.7	6.1±0.7	16.5±3.7	22.1±0.5	19.4±5.0	18.5±0.1
chest	AGKH	160.0±0.0	8.5±1.8	12.7±2.7	40.6±8.3	35.1±12.5	81.4±32.0	22.6±0.4
	BMC	90.0±0.0	10.0±0.0	10.0±0.0	30.0±.0.0	30.0±0.0	60.0±0.0	48.1±0.0
	KCMC	280.0±0.0	10.0±0.0	10.0±0.0	—	31.0±2.8	31.0±2.8	16.9±0.0
	MMH	124.5±0.0	10.0±0.0	15.0±0.0	34.0±2.4	35.5±2.1	76.9±33.3	35.2±0.0
	MNH	280.0±0.0	8.3±2.6	8.3±2.6	25.5±3.1	25.5±3.1	41.7±11.6	17.8±0.2
	RMC	60.0±0.0	10.0±0.0	10.0±0.0	20.0±0.0	19.5±0.7	39.5±0.7	58.4±0.0
	SHMH	375.0±0.0	10.0±0.0	10.0±0.0	22.0±1.4	22.5±2.1	44.5±3.5	25.1±0.0
	TMJH	165.0±0.0	10.0±0.0	10.0±0.0	29.4±3.2	30.0±2.7	59.4±5.4	18.9±0.0
abdomen	AGKH	161.2±4.9	9.3±1.6	14.0±2.4	40.2±9.8	35.6±12.5	102.9±38.0	25.6±0.6
	BMC	120.0±40.0	9.4±1.3	9.4±1.3	33.3±8.7	36.0±12.7	63.9±14.8	47.9±0.0
	KCMC	310.0±42.4	10.0±0.0	10.0±0.0	—	34.5±13.4	34.5±13.4	17.1±0.1
	MMH	124.7±0.0	10.0±0.0	15.0±0.0	14.0±2.0	14.0±1.9	41.0±11.5	35.2±0.0
	MNH	280.0±0.0	10.0±0.0	10.0±0.0	24.9±2.7	25.1±2.7	47.9±8.6	17.9±0.0
	RMC	60.0±0.0	10.0±0.0	10.0±0.0	22.5±2.1	24.0±5.7	46.5±7.8	58.4±0.0
	SHMH	355.0±51.2	10.0±0.0	10.0±0.0	28.8±10.3	30.1±10.0	55.6±21.4	25.1±0.0
	TMJH	180.0±0.0	8.0±0.0	12.0±0.0	30.6±8.3	31.9±9.7	76.6±21.6	18.7±0.0
l/spine	AGKH	480.0±0.0	3.0±0.0	3.0±0.0	42.2±9.0	—	12.7±2.7	26.3±0.1
	BMC	200.0±0.0	3.0±0.0	3.0±0.0	27.0±5.0	—	8.1±3.4	48.4±0.0
	KCMC	440.0±39.7	3.0±0.0	3.0±0.0	23.1±4.6	—	7.0±1.4	16.6±0.2
	MMH	200.0±0.0	3.0±0.0	4.0±0.0	50.4±3.3	—	20.1±1.3	36.3±0.0
	MNH	310.0±37.1	3.0±0.0	3.0±0.0	38.6±16.0	—	11.4±4.9	17.0±0.3
	RMC	60.0±0.0	5.0±0.0	5.0±0.0	27.3±1.5	—	13.7±0.8	58.4±0.0
	SHMH	495.0±95.3	3.0±0.0	3.0±0.0	32.3±6.4	—	9.7±1.9	23.9±0.0
	TMJH	360.0±0.0	3.0±0.0	5.0±0.0	34.8±6.0	—	17.2±3.0	18.4±0.0
pelvis	AGKH	160.0±0.0	8.7±2.2	13.1±3.3	26.6±8.7	25.7±11.5	56.1±26.7	25.5±0.5
	BMC	100.0±0.0	10.0±0.0	10.0±0.0	30.0±0.0	30.0±0.0	60.0±0.0	47.9±0.0
	KCMC	230.0±70.7	10.0±0.0	10.0±0.0	—	38.5±3.5	38.5±3.5	16.8±0.1
	MMH	157.7±0.0	10.0±0.0	12.0±0.0	32.0±4.3	—	39.4±5.2	34.9±0.0
	MNH	220.0±0.0	10.0±0.0	10.0±0.0	27.0±3.0	27.0±3.0	54.0±4.2	17.9±0.0
	TMJH	246.7±57.7	8.7±1.2	11.3±1.2	36.3±4.0	36.3±4.0	81.4±12.0	18.8±0.1

It is further evident from Table 2 that the scan length (calculated from slice thickness, table increment, and number of slices) among scanners varied up to a factor of 1.5, 2.6, 3.0, 2.9, and 2.1 for head, chest, abdomen, lumber spine, and pelvis CT examination, respectively. These variations as also observed elsewhere were largely caused by different scanning protocols (e.g., slice thickness, number of slices, and use of contrast) used by the hospitals.^(^ ^10^ ^,^ ^13^ ^,^ ^18^ ^)^ Large scan length was mainly attributed to the use of contrast media, since in most cases this procedure involves a repeated scan of the same scan length. Another scanning parameter that is not shown in Table 2 is gantry tilt, which was often done for head and lumbar spine. For about 60% of CT examinations of head and lumbar spine for all hospitals, the gantry was tilted to have slices parallel to supraorbitomeatal (SOM) and intravertebral disk lines, respectively. However, this parameter was not used as input to the software since the software does not take into account gantry tilt.^(18)^ Lack of this capability inevitably makes accurate estimation of organ dose difficult to achieve. Further, not taking angulation into account is expected to exaggerate dose estimation, especially dose to the lens of the eyes. Alternatively, the entrance surface dose by direct measurements on patients using TLDs could accurately estimate the dose to this superficial organ. The angulation of gantry parallel to the SOM has proved to some extent to protect the lens of eyes from CT examination of the brain.^(^ ^3^ ^,^ ^18^ ^)^ Apart from angulation of the gantry, no hospital in this study was found protecting superficial radiosensitive organs such as the lens of the eyes, thyroid, and breast, which are rarely the organs of clinical concern in the CT examinations.^(^ ^3^ ^,^ ^7^ ^)^ It has been shown that by using radioprotective material such as bismuth for shielding superficial radiosensitive organs, the radiation dose to the patient can be reduced significantly without loss of image quality.^(7)^

### B. CT dose measurements

Of interest in this study is the determination of organ doses. However, as described earlier, this quantity cannot be determined without the knowledge of normalized $\text{CTDI}{(}_{n}\text{CTDI})$. The CnTDI to mAs for CT scan under typical operation conditions for each scanner model were determined as described earlier from measurements of CTDI in air ${(}_{n}{\text{CTDI}}_{\text{air}})$. The results of the measurements of CnTDIair for CT scans under typical operation conditions for each scanner are presented in Fig. 1. Derived estimates of CTDI normalized to the exposure setting, CnTDIair(mGy (mAs)−1) have been analyzed by scanner type, nominal slice width, applied potential, and focus‐to‐axial distance. Overall, individual measurements revealed that a variation as large as a factor of $2.8\;(0.17\;\text{to}\;0.48\;\text{mGy}\;{\left(\text{mAs}\right)}^{-1})$ was obtained between the mean values of CnTDIair per scanner model.

It is interesting to observe in Fig. 1 that similar makes but different models of scanners can have such large deviations of CnTDIair and, hence, organ doses. The relatively high values shown for Tomoscan M‐EG are probably a function of their short focus‐to‐axial distance as shown in Table 1. This is due to the fact that radiation intensity varies as the inverse of the squared distance between the source of the radiation and the object (patient). As a result, if all other scanning parameters are held constant, the scanner with the shorter distance between the X‐ray tube focal spot and the isocenter of the gantry aperture can produce more radiation exposure than the long geometry scanner. On the other hand, the variation of source‐to‐detector distance among scanners might affect the image quality. This is due to the fact that image noise in CT is known to be inversely proportional to the square root of the number of photons received by the detector, whereas the number of photons (dose) is inversely proportional to the squared distance between the source of the radiation and the detector. As a result, if all other scanning parameters are held constant, the scanner with the long distance between source and detector can have higher image noise than the short geometry scanner. The difference in the case of Somatom AR. Star and Somatom Plus 4 is probably the result of the use of different kV and focus‐to‐axial distances. The CnTDIair are also presented in Fig. 1 for different scanners using different slice thicknesses. From this figure, it is evident that the effect of slice thickness on CnTDIair and, hence, organ dose is marginal for some scanners (i.e., Somatom Plus 4 and Tomoscan SR 4000) and significant for others (i.e., CT/e and Somatom AR. Star).

### C. Doses to selected organs

**Figure 3:**
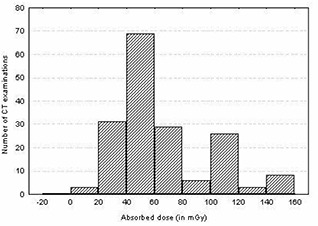
Histogram of dose distribution to the lenses of the eyes for all CT examinations of the head

**Figure 4:**
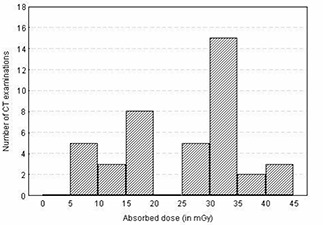
Histogram of dose distribution to the breast for all CT examinations of the chest

**Figure 5:**
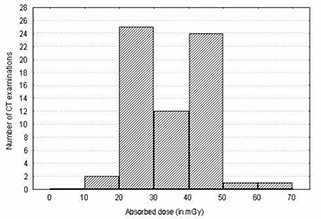
Histogram of dose distribution to the stomach for all CT examinations of the abdomen

**Figure 6:**
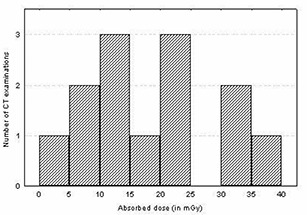
Histogram of dose distribution to the gonads for all CT examinations of the pelvis

The total mean organ doses of selected organs were determined as described earlier for different examinations including the head, chest, abdomen, lumbar spine, and pelvis using patient exposure parameters specific to hospitals and scanners used. The results of estimated total mean organ doses for selected organs per examination are presented in Fig. 2 and Table 3. It is evident from the table that large variations of organ dose exist within and among hospitals. For instance, the mean organ doses per hospital for the eye lens (for head) and breast (for chest examination) varied up to a factor of 2.7 and 2.4, respectively, while for the stomach (for abdomen) and ovary (for pelvis), the variation was up to a factor of 2.1 and 2.4, respectively. The observed wide variation of organ doses in Figs. 3 to 6 for all CT examinations of head, chest, and abdomen, respectively, is an indication that different scanning parameters (i.e., kV, mAs, scan length, and use of contrast media) used in hospitals have a significant influence on organ dose determinations. It was observed that MMH always had the highest organ doses, followed by the TMJH. MMH, for example, uses CT scanners operated at a higher voltage (130 kV), while the rest use 120 kV. Other factors include a contribution of use of larger scan length, exposure settings (mAs), and contrast media. The use of contrast media by some hospitals was done as a routine procedure without clinical justification of using it. In a few hospitals, such as AGKH and TMJH, the radiologist was consulted before IV administration of contrast. KCMC appears to consistently have the lowest organ doses for the chest (Table 3(b)), abdomen (Table 3(c)), and pelvis (Table 3(e)). This was expected because the protocols used exclude scan sequence without contrast. The observed high mean organ doses at TMJH for pelvis examination relative to other hospitals is an indication that large scan length has a significant impact on organ dose.

**Table 3:** Summary of mean organ doses per hospital for each CT examination

	*Mean organ doses per hospital (mGy)*							
Selected organ	AGKH	BMC	KCMC	MMH	MNH	RMC	SHMH	TMJH
(a) Head								
RBM^a^	2.4±1.0	3.9±1.5	3.4±1.1	5.8±1.7	3.3±0.5	2.1±0.3	2.9±0.5	4.5±1.1
brain	37.5±16.1	62.6±23.4	52.0±1.0	84.4±22.6	51.2±9.5	32.5±4.8	48.4±8.5	72.1±16.5
eye lens	41.0±21.0	75.0±28.1	57.0±10.1	110.3±35.2	53.6±11.1	40.2±6.3	92.8±9.6	92.5±21.0
Thyroid	1.6±0.8	2.4±1.0	2.5±1.0	4.7±1.5	2.3±0.4	1.5±0.4	2.3±1.1	3.0±1.0
(b) Chest								
RBM^a^	7.8±3.2	12.4±4.4	7.0±0.5	10.7±6.0	10.3±2.6	7.9±0.1	11.4±0.8	11.6±1.0
lung	20.1±6.3	44.0±7.9	24.0±0.0	25.3±10.1	34.6±12.7	31.5±3.5	36.0±5.7	38.9±2.1
breast	14.8±0.0	36.0±8.1	20.0±0.0	21.0±8.1	29.6±17.1	29.5±0.7	31.0±1.4	33.1±1.0
Thyroid	21.5±0.0	9.0±2.7	4.9±0.0	4.6±0.0	8.7±2.1	3.5±1.9	12.1±1.0	9.3±1.2
esophagus	23.3±0.0	50.0±5.5	27.0±0.0	27.0±0.0	52.0±5.5	42.5±0.7	68.0±0.0	43.6±0.8
(c) Abdomen								
liver	26.1±10.1	42.8±5.2	21.0±1.4	28.0±4.2	41.6±6.2	33.0±1.4	40.5±10.2	30.7±6.5
stomach	28.5±10.9	46.4±6.0	22.5±0.7	28.0±3.5	42.4±6.5	36.0±0.0	42.7±11.6	32.3±6.9
pancreas	24.9±9.8	39.6±4.9	18.5±0.7	26.0±6.0	37.9±5.5	31.0±1.4	44.7±13.5	28.7±6.2
spleen	26.8±10.4	42.1±5.3	20.5±0.7	28.0±1.9	41.5±6.0	33.0±2.3	37.9±9.4	30.5±6.2
kidney	32.0±12.6	51.6±7.5	25.0±0.5	30.0±5.0	44.6±7.2	39.0±1.7	47.3±13.6	47.3±7.2
adrenal	26.4±10.4	40.1±5.2	18.5±0.7	26.0±2.8	40.3±5.6	32.0±1.4	49.4±14.3	29.7±6.1
gland								
(d) Lumbar spine								
large	32.4±2.4	29.0±9.2	9.6±3.0	21.1±1.1	9.4±3.8	11.5±0.5	17.9±0.0	17.4±7.3
intestine								
small	32.9±3.5	28.0±6.2	10.5±2.7	22.4±1.3	11.9±3.9	11.5±0.7	18.1 ±0.0	18.0±6.2
intestine								
colon	17.8±4.6	8.8±3.2	8.3±2.3	18.3±1.4	8.5±2.3	5.2±0.6	9.3±3.4	12.0±2.8
gonads	8.2±10.8	7.4±2.6	11.7±6.9	11.5±11.2	8.1±5.7	9.0±1.8	2.6±3.1	12.6±10.4
uterus	28.6±7.3	6.2±2.1	15.6±8.2	27.3±2.8	11.9±5.9	8.7±3.1	14.4±6.5	19.0±5.8
(e) Pelvis								
ovaries	21.0±1.0	23.0±0.0	16.4±5.1	21.8±0.0	32.2 ±0.0	—	—	39.7±16.2
bladder	26.0±13.5	26.0±0.0	19.5±6.4	25.0±0.0	38.0±0.0	—	—	38.6±24.7
uterus	23.4±11.8	26.0±0.0	17.5±4.9	23.0±0.0	34.0±0.0	—	—	42.7±18.1
Testis	11.2±16.0	5.0±0.0	—	7.6±0.0	7.0±0.0	—	—	16.7±11.4

a RBM=red bone marrow.

Despite the fact that CT scanning protocols were highly standardized for most of the hospitals, wide variation doses among patients up to a factor of 3 were also observed within the hospitals. For instance, the doses to the eye lenses for TMJH and KCMC varied from 52.0 mGy to 130.6 mGy and from 30.5 mGy to 109.4 mGy, respectively, while for MNH and MMH, the doses varied from 30.8 mGy to 82.0 mGy and from 49.5 mGy to 149.0 mGy, respectively. These variations were largely attributed to variation of clinical indications among patients, use of contrast, and number of slices used depending on patient size. It was further observed that organ dose distributions (shown in Figs. 3 to 6) for a given examination vary to an order of magnitude with a broad and relatively flat distribution. Such a wide spread in organ doses observed in this study is comparable to similar surveys performed in other countries.^(^ ^6^ ^,^ ^9^ ^,^ ^10^ ^,^ ^13^ ^,^ ^18^ ^,^ ^19^ ^)^ For instance, the wide spread of doses to the lenses of the eyes in this study ranged from 12.8 mGy to 149 mGy (median 53.8 mGy), while in Australia, Norway, and Japan the spread ranged from 10 mGy to 160 mGy (median of 61 mGy), 39.1 mGy to 108.6 mGy, and 8.7 mGy to 47.2 mGy, respectively.^(^ ^13^ ^,^ ^18^ ^,^ ^19^ ^)^ The wide spread of organ dose distribution is again influenced by variation of techniques used among scanners.

### D. Comparison with other studies

To facilitate the comparison between nations, the mean values of number of slice (*n*) and mean organ doses of selected organs per examination in this study and from reported values from the literature for the United Kingdom, Germany, the Netherlands, and Japan have been presented in Table 4.^(^ ^6^ ^,^ ^9^ ^,^ ^10^ ^,^ ^19^ ^)^ It is clear from the table that with the exception of values reported from Japan, the mean organ doses per given examination were mostly comparable with those from other studies. For instance, the variation of organ doses between this study and reported values from the Netherlands and the United Kingdom mostly varied by up to a factor of 1.0 and 1.7, respectively, while for Germany and Japan, the organ doses mostly varied by up to a factor of 2.3 and 3.0, respectively. On the one hand, the higher organ doses observed in this study relative to that reported from Japan might be attributed to the different method used for estimation of organ doses, whereby that of Nishizawa et al.^(19)^ used TLD in a female Rando Alderson phantom. In addition, the higher organ doses in this study relative to those of Japan might also have been attributed to the fact that Nishizawa et al. estimated the organ dose from a single scan at a fixed number of slices for a given examination, while in this study organ doses were estimated from an examination with either one scan (i.e., without or with contrast) or with two scans, one with and one without contrast.

**Table 4:** Comparison of mean organ doses in this study and other studies

		*This study*		*UK* ^(10)^		*Germany* ^(6)^		*Netherlands* ^(9)^		*Japan* ^(19)^	
CT examination	Selected organ	*n* ^a^	*D* (mGy)	*n* ^a^	*D*(mGy)	*n* ^a^	*D*(mGy)	*n* ^a^	*D*(mGy)	*n* ^a^	*D*(mGy)
head	RBM^b^	42	3.5±1.6	25	2.7		—		—	9	1.5
eye lens	42	63.9±32.6	—	—	24	24.8		—	9	22.4
thyroid	42	2.5±1.3	25	1.9		—		—	9	0.6
chest	RBM^b^	60	9.9±2.8	34	5.9	—	—	28	10	16	5.7
lung	60	31.5±10.6	34	22.4	49	20.5	28	37	16	19.6
breast	60	26.1±10.8	34	21.4	49	22.6	28	32	16	15.9
thyroid	60	12.3±8.5	34	2.3	—	—	28	7	16	1.9
abdomen	liver	61	34.1±10.3	31	20.4	32	15.0	31	35.5	13	27.8
stomach	61	35.6±10.7	31	22.2	32	15.4	31	38.5	13	26.9
pelvis	ovary	62	24±17.1	25	22.7	52	14.9		—	12	15.1
bladder	62	28.8±21.2	25	23.2	52	16.1		—	12	10.6
uterus	62	26.5±18.6	25	25.5	52	14.6		—		—
testis	62	12.5±19.9	25	1.7	—	—		—	12	1.0

a *n* is the number of slices (with and without contrast).

b RBM=red bone marrow.

On the other hand, the differences in organ doses between this study and those reported in the literature for the United Kingdom, Germany, and the Netherlands with NRPB organ dose conversion factors were mainly attributed to the variation in CT scanning protocols (i.e., kV, mAs, slice thickness, number of slices, use of contrast media, etc.) and type of scanners used. For instance, the average number of slices used in this study was higher by factor of 1.7 to 2.5, 1.2 to 1.9, and 2.1 than the average number of slices reported in the literature for the United Kingdom, Germany, and the Netherlands, respectively. This further suggests that there exists potential for dose reduction in Tanzanian hospitals through decreasing numbers of slices (hence scan length). Moreover, the mean doses for selected organ per examination in this study were found to be comparable to those reported by Olerud of Norway.^(18)^ For example, the mean organ dose in this study for the lenses of the eyes, red bone marrow (for head), ovary (for pelvis), and testis (for pelvis) were 63.9 mGy, 3.5 mGy, 24.0 mGy, and 12.5 mGy, respectively, while for Norway they were 80.9 mGy, 3.2 mGy, 26.0 mGy and 7.0 mGy, respectively.

In view of the observed causes of organ dose variations and similar experience observed elsewhere,^(^ ^6^ ^,^ ^10^ ^,^ ^16^ ^–^ ^18^ ^)^ further studies on the optimization of organ doses to patients undergoing CT examinations are needed. There are a number of observed parameters that greatly need optimization:

1. The first is the minimization of the number of slices (hence scan length) as much as possible, without missing any vital anatomical region. Several studies have recommended that with the reduction of irradiation volume depending on body region being scanned, radiation dose to patients can be significantly reduced.^(^ ^3^ ^,^ ^9^ ^,^ ^10^ ^,^ ^13^ ^,^ ^17^ ^,^ ^18^ ^)^
2. The second is minimization of tube current (mA) based on indication of study. Some studies have already revealed that adjustment of mA based on indication of study dose to patient can be reduced to 50% without significantly affecting the image quality.^(^ ^20^ ^,^ ^21^ ^)^
3. The third approach is through modulation of exposure parameters (i.e., kV, mA, exposure time, and slice thickness) based on patient size and age, while maintaining a constant contrast to image‐to‐noise ratio. Some studies have demonstrated that by adjusting the exposure parameters based on patient age, weight, or transverse diameter of the body part imaged, patient radiation doses can be reduced significantly.^(^ ^17^ ^,^ ^21^ ^,^ ^22^ ^)^
4. Another possible method is through use of contrast media only to optimize diagnostic yield. As mentioned earlier, use of contrast media by some of the hospitals was done as a routine procedure without clinical justification of using it. In only a few hospitals (AGKH, TMJH, and MMH) were the radiologists consulted before the IV administration of contrast.
5. The last possible method is the use of radioprotective materials for protecting superficial radiosensitive organs such as the lenses of the eyes, thyroid, and breast. It has been demonstrated that in‐plane bismuth shields could significantly reduce absorbed dose to these superficial organs without loss of image quality.^(^ ^3^ ^,^ ^7^ ^,^ ^8^ ^)^

However, in order to achieve the above optimization strategies, there is a great demand to educate CT personnel on the effects of scan parameter settings on radiation dose to patients and image quality required for accurate diagnosis.

## IV. CONCLUSIONS

The assessment of radiation dose to selected radiosensitive organs of patients undergoing CT examinations in Tanzania was investigated. In this study, large variations of radiation dose to various organs were observed. Different scanning protocols used among hospitals and variation in equipment design among manufacturers and models were responsible for these variations. The mean organ doses in this study were mostly comparable to and slightly higher than reported values from the United Kingdom, Germany, the Netherlands, Norway, and Japan. The main contributor for this difference was the use of a larger scan length in Tanzania than that used in some of these countries. The large observed variations of organ doses among hospitals and relatively high organ doses in Tanzanian hospitals call for the need to optimize CT scanning protocols. This can be achieved through optimal selection of scanning parameters based on indication of study, body region of interest being scanned, and patient size. In addition, further studies should be done to investigate the potential for using radioprotective materials to protect superficial radiosensitive organs.

## ACKNOWLEDGMENTS

The authors acknowledge the National Institute for Medical Research for approval of their our proposal. We sincerely appreciate the financial and equipment support from the Tanzania Atomic Energy Commission. We would like to thank the authorities of the hospitals included in the survey for allowing us to use their CT facilities. We would further like to thank the following radiographers: Messrs. P. Masue (KCMC), B. Lema (MNH), P. Mbosoli (BMC), M. Msuya (MMH), D. Matembo (RMC), E. Kamala (SHMH), M. Mallya (AGKH), and B. Nsheto (TMJH) for their technical support at CT facilities.
